# An Experimental Rat Model Study: Is There Any Effect of Syringic Acid on Ischemia-Reperfusion Injury in Priapism?

**DOI:** 10.7759/cureus.45475

**Published:** 2023-09-18

**Authors:** Kubilay Sarikaya, Engin Kölükçü, Velid Unsal, Süleyman Özdemir

**Affiliations:** 1 Urology, Health Sciences University Ankara Etlik City Hospital, Ankara, TUR; 2 Urology, Tokat Gaziosmanpaşa University School of Medicine, Tokat, TUR; 3 Biochemistry, Faculty of Health Sciences and Central Research Laboratory, Mardin Artuklu University, Mardin, TUR; 4 Pathology, Faculty of Medicine, Gaziosmanpasa University, Tokat, TUR

**Keywords:** cavernosal tissue, rat model, oxidative stress, corporal tissue, priapism

## Abstract

Purpose

The purpose of this research is to examine the impact of syringic acid on ischemia-reperfusion injury in cavernosal tissue, utilizing a rat model of induced priapism.

Materials and methods

A total of 24 rats were allocated into three groups. Group 1 was designated as the control group, while Group 2 underwent ischemia-reperfusion injury assessment using the priapism model. Group 3 underwent the same procedures as Group 2, with the addition of intraperitoneal administration of syringic acid (100 mg/kg) 60 min after priapism initiation. All rats underwent penectomy, and sufficient blood samples were collected. Histopathological assessment of penile cavernosal tissue involved grading tissue damage, inflammation, vasocongestion, desquamation, and edema on a scale of 0-3 (0: normal, 1: mild, 2: moderate, 3: severe).

Result

Significant differences were observed among the three groups in terms of IL-1 beta and TNF-alpha levels (p=0.001 and p<0.001, respectively). IL-1 beta and TNF-alpha levels in Group 2 were found to be significantly higher than Group 3 (p=0.003 and p=0.004). There was also a significant difference among the three groups in terms of median MDA levels (p<0.001). Furthermore, the median MDA level in Group 2 was found to be significantly higher than that in Group 3 (p<0.001). While significant differences were observed among the three groups in terms of median SOD and GSH-px levels, no significant difference was found among the groups in terms of median PC levels (p=0.004, p= 0.048, and p=0.159, respectively). In direct microscopic examination, a significant improvement in pathological scores was noted in Group 3 compared to Group 2 (p<0.001).

Conclusion

Syringic acid demonstrated protective properties against ischemia-reperfusion injury caused by priapism in cavernosal tissue.

## Introduction

Ischemic priapism represents a urologic emergency medical situation in the urological area. This condition has been characterized as an extended, painful erection lasting for more than four hours, which occurs without any sexual desire or stimulation [1]. Priapism is categorized into three main types: ischemic priapism (also called low flow or veno-occlusive priapism), nonischemic priapism (referred to as high flow or arterial priapism), and stuttering priapism (involving intermittent, recurrent ischemic episodes). The prevalent form is ischemic priapism, which stems from reduced blood flow, and this particular type comprises approximately 95% of all reported instances [2]. Due to its connection with venous outflow obstruction and stagnant blood flow, priapism leads to substantial tissue hypoxia and acidosis. This progression leads to ischemia and fibrosis in the smooth muscle of the cavernosal tissue, ultimately resulting in the onset of profound and permanent erectile dysfunction (ED) [3]. Although the reperfusion of ischemic tissue is crucial for initiating reparative mechanisms in the body, it has been shown that reperfusion can actually worsen the damage caused by ischemia. This is primarily due to the release of reactive oxygen species (ROS) into the systemic circulation. Oxidative damage arises from an excessive generation of ROS and free radicals, overwhelming the body's inherent antioxidant defense mechanisms [4]. The process of oxidative damage results in an increase in the levels of malondialdehyde (MDA) and protein carbonyl (PC) within the tissue. These substances serve as end products arising from lipid peroxidation and protein oxidation, respectively [5].

The standard approach to dealing with priapism involves utilizing penile aspiration and administering alpha-adrenergic agents. In cases where medical intervention proves inadequate, diverse surgical approaches are explored [6]. Numerous antioxidant agents have been utilized in previous studies to demonstrate their protective effects against oxidative injury in various organ systems, particularly in the context of ischemia-reperfusion injury [7,8]. Syringic acid is a naturally occurring phenolic acid known for its inherent antioxidant properties that enable it to effectively neutralize free radicals [9]. Syringic acid's role as an antioxidant can be explained by its electron-donating ability and the stability of its phenoxy radical intermediate. These characteristics allow syringic acid to effectively neutralize and scavenge free radicals, thereby protecting cells and tissues from oxidative damage [10]. On the other hand, none of the studies we mentioned have shown that syringic acid has serious side effects.

This study aimed to investigate the effects of syringic acid on cavernosal tissue subjected to ischemia-reperfusion injury resulting from priapism. To our knowledge, this study is the first in the contemporary medical literature to explore the potential impact of syringic acid in a rat model of priapism. This study’s goal is to highlight the therapeutic potential of syringic acid in managing priapism-associated ischemia-reperfusion injury and its implications for future treatments.

## Materials and methods

The protocol of this experimental study was approved by the Tokat Gaziosmanpaşa University Animal Studies Ethics Committee (2022/ HADYEK-18). The study involved the use of 24 adult male Wistar-albino rats, aged between 7.5 and 8.5 months old, with weights ranging between 300 g and 350 g. The rats were maintained within the laboratory setting in compliance with institutional protocols and the Guide for the Care and Use of Laboratory Animals by the National Research Council. They were housed under typical vivarium conditions in a room under controlled climate conditions, maintaining a temperature range of 18-22°C, humidity levels between 40-60%, and a 12-hour light/dark cycle. The rats had ad libitum access to water and were fed standard rodent chow. The rats were classified into three groups: Groups 1-3, each consisting of eight rats. Group 1 functioned as the control group, Group 2 was designated as the ischemia-reperfusion group, and Group 3 was assigned as the treatment group. All surgical procedures were performed with the proper level of anesthesia and under sterile conditions. For this purpose, xylazine hydrochloride (Rompun 2%, Bayer, Turkey), an anesthetic agent with sedative and muscle relaxation properties, was administered intraperitoneally at a dose of 10 mg/kg. Additionally, ketamine hydrochloride (Alfamine 10%, Ege Vet, Turkey), which provides dissociative anesthetic effects, was administered intraperitoneally at a dose of 50 mg/kg. These anesthetic agents were administered to ensure the rats' comfort and maintain sterility during the surgical procedures.

In Group 1, the rats were allocated to the control group, and a single penectomy was conducted solely within this group. The penile tissues obtained were sent to the pathology laboratory to undergo histopathological examination. Moreover, blood samples were obtained from the inferior vena cava of these rats for biochemical analysis. Priapism was induced in Group 2 using the method described by Sanli et al [11]. To create a vacuum erection device, the tip of a 5-cc syringe was adapted to suit its attachment to the base of the flaccid penis. Before applying the vacuum to the penis, a 2-mm wide constriction band was placed around the tip of the vacuum erection device. After positioning the syringe tip at the base of the penis and gently pulling it back, an erection was induced in the rat's penis, attaining a sufficient level of erection. Once the erection was deemed satisfactory, the constriction band slipped off the syringe and was placed at the base of the penis. After the constriction bands were removed, the rats were allowed to rest for 1 h to evaluate ischemia-reperfusion injury. At the conclusion of the procedure, a penectomy was performed to enable histopathological examination, and blood samples were procured from the rats' inferior vena cava for subsequent biochemical analysis. In Group 3, the rats underwent procedures similar to those in Group 2. Additionally, Group 3 rats received a single dose of syringic acid (100 mg/kg) administered intraperitoneally 30 min after the induction of priapism. Syringic acid was obtained from Sigma-Aldrich for use in the study. It was dissolved in dimethyl sulfoxide (DMSO) and then diluted with 0.9% physiological saline solution just before administration. Following the identical 1-hour reperfusion interval, a penectomy was performed, and blood samples were collected to evaluate the effects of syringic acid on the rats within Group 3. Corpus cavernosum tissue samples were used for pathological examination after penectomy in all groups.

Biochemical evaluation

Measurement of Malondialdehyde (MDA) Levels

MDA is the end product of lipid peroxidation and serves as a commonly employed marker for assessing oxidative stress. It reacts with thiobarbituric acid (TBA) in an acidic environment at elevated temperatures, resulting in the formation of a pink-colored compound. This reaction is widely utilized for quantifying oxidative stress levels. The optical density of this compound was assessed at a wavelength of 532 nm, and the MDA level was determined from this measurement. The results were determined by referencing a standard graph constructed using serial dilutions of standard 1,1,3,3-tetraethoxypropane. The MDA level was quantified and expressed in units of micromoles per liter (μmol/L) [12].

Measurement of Superoxide Dismutase (SOD) Activity

The underlying principle of this experiment relies on the generation of a violet-colored formazan compound. This compound is produced through the action of the superoxide radical, which is generated using the xanthine-xanthine oxidase system. The generated superoxide radical then reduces the nitroblue tetrazolium (NBT) compound present in the environment, resulting in the formation of a distinctive violet-colored formazan compound. When measured using a spectrophotometer, this compound exhibits its highest absorption at a wavelength of 560 nm. When serum is introduced into the reaction environment, the SOD enzyme functions to eliminate the superoxide radicals generated from the surroundings. This action effectively inhibits the reduction of NBT in a manner directly proportional to the enzyme’s activity. Enzyme activity was determined by comparing the absorption value obtained when SOD was introduced to the reaction environment with the baseline absorbance from a control experiment in which the enzyme was not added. The activity of SOD was quantified and expressed in units per liter (U/L) [13].

Measurement of Glutathione Peroxidase (GSH-px) Activity

The enzyme glutathione reductase facilitates the conversion of oxidized glutathione (GSSG) back to its reduced form GSH, simultaneously utilizing nicotinamide adenine dinucleotide phosphate (NADPH) and converting it to nicotinamide adenine dinucleotide (NADP). When analyzed with a spectrophotometer, it induces NADPH absorption at a wavelength of 340 nm. The decrease in absorption resulting from the conversion to NADP enables the assessment of GSH-px activity. GSH-px activity was quantified and expressed in units per liter (U/L) [14].

Measurement of Penile Tissue Protein Carbonyl (PC) Levels

Carbonyl content was assessed spectrophotometrically using a Cintra 10 E spectrophotometer from Austria. This determination relied on the reaction between carbonyl groups and 2,4-dinitrophenylhydrazine to produce 2,4-dinitrophenylhydrazone [15]. The outcomes were reported in units of nanomoles per milliliter (nmol/mL) for penile tissue.

Measurement of Tumor Necrosis Factor-Alpha (TNF-Alpha) and Interleukin-1 Beta (IL-1 Beta) Levels

The ELISA kits were provided by Atlas Biotechnology (Ankara, Turkey). TNF-alpha and IL-1 beta levels in the serums were assessed using these kits, following the manufacturers’ instructions. The measurement was conducted using the Multiskan™ FC Microplate Photometer device. The levels of TNF-alpha and IL-1 beta were quantified and reported in picograms per milliliter (pg/mL), while IL-6 levels were expressed in nanograms per milliliter (ng/mL).

Histopathological evaluation

The penile tissues from the rats were preserved in a 10% buffered formalin solution for a duration of 2 days. Subsequently, the samples were sectioned to a thickness of 4 μm using a microtome and subjected to staining with hematoxylin and eosin solutions. This staining process followed the initial grossing and preparation of the tissue samples. Tissue slides were examined using an upright light microscope (Nikon Eclipse E600). The evaluation of tissue slides involved assessing and assigning scores in categories such as vasocongestion, inflammation, desquamation, and edema. A semiquantitative scoring system, similar to the method utilized by Sentürk et al. [16], was adopted to assess histopathological changes. These parameters were graded on a scale from 0 to 3, where 0 represented normal, 1 indicated mild alteration, 2 signified moderate alteration, and 3 denoted severe alteration.

Statistical Analysis

All statistical analyses were performed using SPSS 22.0 software (IBM Corp., Armonk, NY) on the Windows platform. A significance level of P<0.05 was considered statistically significant for all tests conducted. The distribution was assessed using the Shapiro-Wilk test. Parametric variables were analyzed using the t-test, one-way analysis of variance (ANOVA), and post hoc Bonferroni analysis. Conversely, nonparametric variables were analyzed using the Mann-Whitney U test and Kruskal-Wallis tests. Data for nonparametric variables were reported as median and minimum-maximum range, and data for parametric variables were presented as mean±standard deviation.

## Results

In this study, proinflammatory cytokines, including TNF-alpha and IL-1 beta, were found to be dramatically significant between the three groups (p<0.001 and p=0.001). While the median TNF-alpha value was 438.5 (385-500) in Group 2, it was found to be median 380 (305-404) in Group 3 (p=0.004). Similarly, the mean IL-1 beta value of Group 2 was 360.87, while this value was 304.75±17.74 in Group 3, and a significant difference was found among the groups (p=0.003). While the mean MDA level was 4.33+0.67 in Group 2, it was detected as a mean of 1.93+0.32 in Group 3, and a significant difference was found between the groups (p<0.001). Additionally, while the median GSH-px level was measured as 625.5 (458-698.9) in Group 2, this value was measured as a median of 641.9 (579-781.1) in Group 3, and no significant difference was found between the two groups (p=0.529) However, in our study, while the median SOD level was 5.55 (4.10-6.90) in Group 2, it was measured as mean 6.20 (5.31-8.10) in Group 3, and no significant difference was found between the groups (p=0.292). Nevertheless, Group 1 displayed a significantly higher median SOD level compared to Group 2 (p=0.003). No significant difference was observed among the three groups in terms of PC levels (p=0.159). The biochemical results of this study are shown in Table 1.

**Table 1:** Comparison of IL-1 beta, MDA, SOD, GSH-px, TNF-alpha, and PC values in three rat groups IL-1 beta: Interleukin-1 beta, MDA: malondialdehyde, SOD: superoxide dismutase, GSH-px: glutathione peroxidase, TNF-alpha: tumor necrosis factor-alpha, PC: protein carbonyl SD: Standard deviation

	Groups	Mean±SD	P value	Post-hoc p values
IL 1 beta	1	280.62±19.79	0.001	1-2: <0.001
(pg/ml)	2	360.87±35.77		1-3: 0.050
	3	304.75±17.74		2-3: 0.003
MDA	1	1.45±0.39	<0.001	1-2: <0.001
(μmol/L)	2	4.33±0.67		1-3: 0.015
	3	1.93±0.32		2-3: <0.001
		Median (min-max)		
SOD	1	7.85 (5.50-9.90)	0.004	1-2: 0.003
(U/L)	2	5.55 (4.10-6.90)		1-3: 0.137
	3	6.20 (5.31-8.10)		2-3: 0.292
GSH-px	1	663.5 (599-800)	0.048	1-2: 0.045
(U/L)	2	625.5 (458-699)		1-3: 0.675
	3	641.9 (559-771)		2-3: 0.529
TNF-alpha	1	327.5 (265-400)	<0.001	1-2: <0.001
(pg/ml)	2	438.5 (385-500)		1-3: 0.208
	3	380 (305-404)		2-3: 0.004
PC	1	499.5 (300-700)	0.159	
(nmol/ml)	2	689.5 (405-799)		
	3	525 (347-755)		

Additionally, a dramatic difference was observed between the groups in the pathological scoring consisting of the sum of inflammation, edema, desquamation, and vasocongestion scores (p<0.001) (Table 2). 

**Table 2:** Comparison of pathological scores in three rat groups * SD: Standard deviation

	Group	mean±SD	Median (min-max)	P value	Post-hoc p values
Pathologic	1	2.25±0.46	2 (2-3)	<0.001	1-2: <0.001
Score	2	7.12±0.64	7 (6-8)		1-3: <0.001
	3	4.87±0.35	5 (4-5)		2-3: <0.001

**Figure 1:**
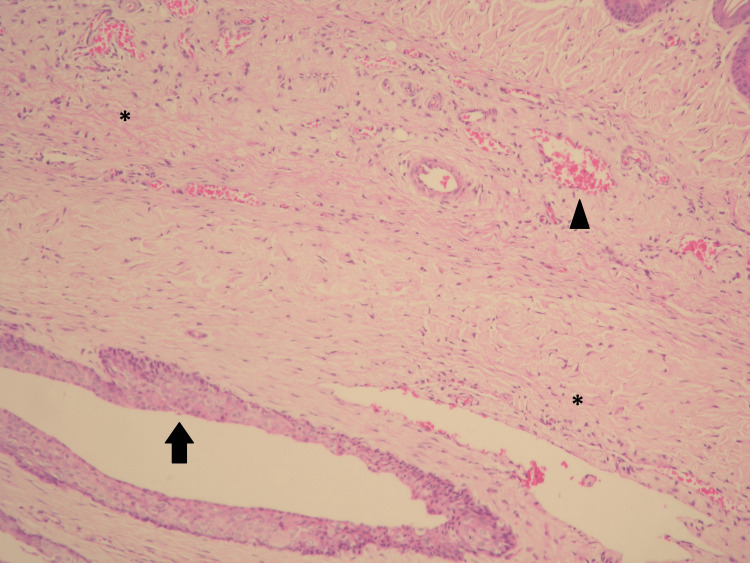
Penile tissue samples from the experimental animals in Group 1 Minimal congestion (arrowhead), minimal inflammatory infiltration (*), and an intact appearance of the epithelial layer (arrow) were examined (magnification=x100).

**Figure 2:**
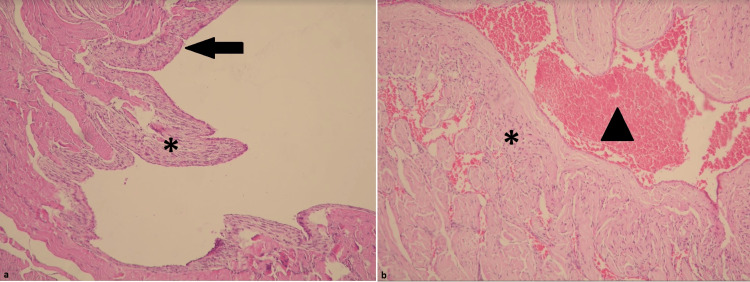
Penile tissue samples from the experimental animals in Group 2 Severe inflammation (*) accompanied by significant epithelial loss, desquamation (arrow), and congestion (arrowhead) were observed (magnification=x100).

Histopathological examination of Group 1 showed minimal congestion, minimal inflammatory infiltration, and an intact epithelial layer, while Group 2 showed severe inflammation, desquamation, and congestion, with almost complete epithelial loss (Figure 1 and Figure 2).

**Figure 3:**
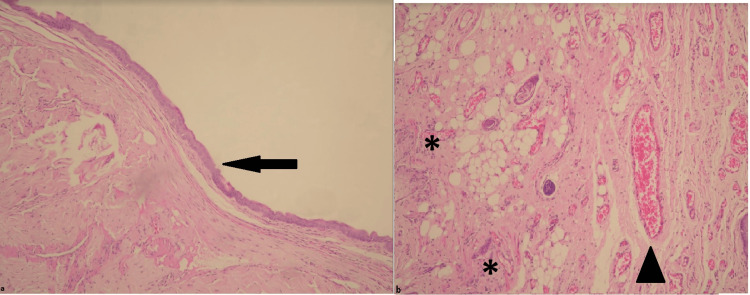
Penile tissue samples from the experimental animals in Group 3 Epithelial thinning, desquamation (arrow), moderate-level inflammation (*), and congestion (arrowhead), were examined (magnification=x100).

Group 3 showed penile tissue samples with moderate epithelial thinning and moderate inflammation, desquamation, and congestion (Figure 3).

## Discussion

Priapism is characterized as a prolonged erection of the penis that persists without sexual stimulation. Ischemic priapism accounts for approximately 95% of all cases [17]. Ischemic priapism represents a critical urologic emergency resulting from an imbalance between vasoconstrictive and vasorelaxant mechanisms. This imbalance leads to the emergence of a closed compartment syndrome within the penis. From a biochemical standpoint, this syndrome is characterized by factors such as hypoxia, hypercapnia, and acidosis [18]. The extent of damage in ischemic priapism is directly linked to its duration. In cases of prolonged and untreated priapism, irreversible harm can transpire into smooth muscle fibers. These fibers are crucial for maintaining proper penile function and dynamics [19]. In cases of ischemic priapism, ultrastructural changes become evident in the smooth muscles after approximately 12 h. Following this, focal necrosis develops around the 24-hour mark. Ultimately, after about 48 hours, a progression toward necrosis and the transformation of fibroblast-like cells occur [20].

The initial treatment strategy for ischemic priapism involves the aspiration of blood from the corpora cavernosa, yielding a success rate of approximately 30%. The secondary treatment approach involves the local intracavernosal administration of sympathomimetics. This method is estimated to achieve an average success rate of up to 80%. The third step in treatment involves surgical shunting procedures [21]. While medical intervention or shunt surgery can ultimately lead to successful detumescence, it is noteworthy that a significant proportion of these individuals experience ED afterward. This incidence of ED is seen in over half of the cases and rises to 90% when the priapism episode lasts for more than 24 hours. Therefore, early treatment of ischemic priapism with appropriate procedures is critical to prevent the loss of erectile function.

Ischemia-reperfusion injury is particularly prominent in this clinical scenario as the penile corpus cavernosa undergoes rapid reoxygenation following the treatment of ischemic priapism. This swift reintroduction of oxygen can result in a substantial degree of reperfusion injury [22]. Throughout tissue ischemia, anaerobic metabolism results in the reduced production of adenosine triphosphate (ATP) and antioxidant agents within cells. Furthermore, the accumulation of lactic acid can contribute to the onset of metabolic acidosis. Additionally, a potential exists for the malfunction of sodium-potassium pumps (Na+-K -ATPase pumps) and calcium pumps (Ca2+ -ATPase pumps) located on the cell membrane. The dysfunction of Na+K+ -ATPase pumps results in the accumulation of sodium within cells and the efflux of potassium from cells. This elevated intracellular sodium level subsequently leads to a reduction in the functionality of sodium-hydrogen exchanger pumps (Na+-H+ pumps). Also, the dysfunction of calcium pumps (Ca2+ -ATPase pumps) located on the endoplasmic reticulum hampers calcium reuptake. This disruption results in the accumulation of hydrogen, sodium, and calcium ions within cells, causing hyperosmolarity. This hyperosmolarity drives the influx of water into the cytoplasm, leading to cellular swelling. The accumulation of hydrogen ions reduces cellular pH, resulting in compromised enzyme activity and aggregation of nuclear chromatin. The dissociation of ribosomes contributes to a decline in protein synthesis. Subsequent to the reperfusion phase, the reintroduction of blood flow to ischemic tissue facilitates the delivery of oxygen through red blood cells. Simultaneously, an elevation in the production of ROS occurs as a result of diminished concentrations of antioxidative agents within ischemic cells. These ROS contribute to oxidative stress, which in turn triggers endothelial dysfunction, DNA damage, and local inflammatory responses. Following the initiation of inflammatory cascades and oxidative stress, the potential for the development of a cytokine storm exists, which can lead to cell death because of extensive damage to cellular structures [23].

Due to all of these factors, it is strongly believed that anti-inflammatory molecules play a crucial role in safeguarding cells against tissue damage during ischemia-reperfusion injury. Maintaining a balanced prooxidant/antioxidant equilibrium is crucial for overall health. The body’s antioxidant defense mechanism is established through the interplay of endogenous and exogenous antioxidants. The antioxidant defense system safeguards the organism by inhibiting the formation of ROS and associated damage as well as facilitating the detoxification processes. Certain substances possess exogenous antioxidant properties that help diminish or entirely counteract the detrimental effects of free radicals. Numerous substances that have been suggested to possess antioxidant properties have been assessed through testing in diverse ischemia-reperfusion models [4,7,16]. Syringic acid (4‐hydroxy‐2,3‐dimethoxybenzoic acid) is a compound well-known for its intrinsic antioxidant properties, enabling it to neutralize free radicals. This attribute can be attributed to its ability to donate electrons and the stability of its phenoxy radical intermediate [10]. The ability of syringic acid to combat radicals has been correlated with its capacity to hinder lipid peroxidation and lower GSH-px levels in the hepatic, renal, and neuronal tissues of diabetic rats [24]. Additionally, recent studies have shown that syringic acid has positive effects in the treatment of human colorectal cancer [25]. The studies have presented biochemical and histopathological evidence that underscores the effectiveness of syringic acid, a powerful anti-inflammatory and antioxidant compound employed in the research, in mitigating ischemia-reperfusion damage in the penile corpus cavernosum.

Numerous prior experimental studies have investigated the impact of ischemia-reperfusion injury on corpus cavernosum tissue. In their experimental priapism study, Kölükçü et al. demonstrated the impact of oxytocin in reducing ischemia-reperfusion injury in the corpus cavernosum [26]. A total of four rat groups, each consisting of 10 rats, were used in their study. According to their study, the control group (n=10) received no intervention. Group 2 involved the establishment and maintenance of a rat model of priapism for a duration of one hour. Group 3 experienced reperfusion for 30 minutes following the priapism period. In Group 4, oxytocin was administered to the rats 30 minutes before reperfusion was initiated following the priapism episode. In this study, Group 4 demonstrated a significant increase in serum GSH-px activities compared to Groups 2 and 3 (p=0.002 and p=0.001, respectively). However, no statistically significant differences were observed among the groups in terms of SOD activities (p>0.05). According to their research, the levels of serum MDA, an end product of lipid peroxidation that rises with oxidative damage, exhibited increases in Groups 2 and 3 compared to the control group. However, multiple comparisons indicated no statistically significant difference (p=0.607). In a comparable study conducted by Evliyaoglu et al., which also investigated corporal MDA levels after priapism, an increase in MDA concentration was noted in all priapic groups when compared to the control group (p<0.001) [4]. Similar to other studies, serum MDA levels were found to be significantly different between the groups in our study. Additionally, the serum MDA level was found to be significantly lower in Group 3, in which 100 mg/kg syringic acid was applied, compared to Group 2, which did not receive any treatment following corporal ischemia. Consistent with the literature, this study also identified a notable difference between the groups concerning the rise in SOD and GSH-px enzyme levels. However, this difference was not deemed statistically significant. This suggests that the observed result may be attributed to the limited number of rats utilized in this study, and statistical significance might be established in studies with a larger rat population.

PC is another marker of oxidative stress, and it can be measured in both serum and tissue, and its levels increase in response to oxidative stress. Uluocak et al. investigated the effects of melatonin on ischemia-reperfusion injury secondary to priapism in a study of 21 rats [27]. In their study, the rats were categorized into three groups, with Group 1 designated as the sham group, Group 2 as the ischemia-reperfusion group, and Group 3 as the treatment group, receiving 50 mg/kg intraperitoneal melatonin 30 min after ischemia. In this study, there was no statistically significant difference observed among the three groups in terms of penile tissue PC values (p>0.05). In another similar study by Kölükçü et al., 24 adult rats were used to investigate the protective effects of dapsone in priapism [28]. In their study, Group 1 was established as the control group. An ischemia-reperfusion injury assessment was conducted using the priapism model in Group 2. Group 3 underwent procedures analogous to those of the rats in Group 2. Furthermore, the rats in Group 3 were intraperitoneally administered 12.5 mg/kg dapsone 30 mins after priapism induction. According to this study, PC values were found to be significantly different in the three groups (p<0.001). Additionally, the PC value in the dapsone treatment group (Group 3) was significantly lower than in the untreated Group 2 (p=0.015). Additionally, in their study, the analysis of serum samples revealed a substantial rise in proinflammatory cytokines, including IL-1 beta and TNF-alpha (p<0.001 and p<0.001). Our study did not reveal any significant difference between the groups in terms of the PC values measured in the penile tissue. However, consistent with the literature data, serum levels of proinflammatory cytokines IL-1 beta and TNF-alpha were found to be significantly different among the three groups in this study. Moreover, the significant reduction in serum IL-1 beta and TNF-alpha values observed in Group 3, which received 100 mg/kg syringic acid treatment, compared to Group 2, which did not receive treatment in our study, lends support to the notion that syringic acid exerts a preventative effect on ischemia-reperfusion injury in penile tissue.

Semiquantitative scales have been used in histopathological evaluations in rat studies examining ischemia-reperfusion injury secondary to priapism. In the study of Karagüzel et al., wherein they showed the effect of dipyridamole on ischemia-reperfusion injury secondary to priapism, a total of 24 rats that were used were divided into four groups [29]. In this study, Group 1 served as the control group. Priapism was induced in Group 2, followed by a cessation of priapism after 1 hour and a subsequent 30-min reperfusion period. In Group 3, 1 mL/kg of dipyridamole was administered intraperitoneally 30 min prior to reperfusion. In Group 4, 10 mg/kg of dipyridamole was administered intraperitoneally 30 min before reperfusion. In their study, blood and penile specimens were collected following the completion of the 30-min reperfusion period. The study focused on examining the sinusoidal area (measured in µm²), tears in the tunica albuginea, and injury parameters within the sinusoidal endothelium of the penis. They concluded that the histopathological assessment showed no notable alterations in terms of the sinusoidal area. However, a reduction in tears was evident in Group 4 compared to Group 2 (p < 0.05). While there was a decrease in endothelial injury in Group 4 compared to Group 2, this difference did not reach statistical significance (p>0.05). However, in this study, histopathological findings were evaluated using a similar semiquantative method described by Sentürk et al., assigning scores to categories such as vasocongestion, inflammation, desquamation, and edema [16]. Similar to the literature data, a significant difference was found between the histopathological score totals of the three groups in this study. Furthermore, the histopathological score of the untreated Group 2 in this study was significantly higher than that of the syringic acid treatment group (Group 3), receiving a dose of 100 mg/kg. This observation holds significant importance as it highlights the protective effect of syringic acid on penile corpora tissue against ischemia-reperfusion injury.

The positive effect of syringic acid on oxidative stress in different tissues has been demonstrated in numerous experimental studies [24,25]. This research is an inaugural instance in the existing literature that demonstrates the beneficial impacts of syringic acid on ischemia-reperfusion injury in penile tissue resulting from priapism. Promising data has been obtained concerning the potential use of syringic acid in standard urological procedures to alleviate the consequences on reproductive systems in cases following priapism. The findings of this study necessitate validation through randomized prospective clinical trials. Conversely, it is essential to undertake more extensive investigations to assess the suitable dosage range and optimal timing of syringic acid administration for the treatment of priapism during subsequent phases. This initial study is anticipated to provide insights for researchers in the field of ED to develop more thorough investigations into the impacts of ischemia and reperfusion on cavernosal tissue. This, in turn, can enhance the utilization of syringic acid in such scenarios and advance the clinical understanding of antioxidant administration implications in the future.

The main limitation of this study is its exclusive concentration on the initial impacts of syringic acid in cases of ischemic priapism, involving the administration of a solitary dose. As a result, it was difficult to explore the extended-term reaction of cavernosal tissue to this pharmaceutical compound. Subsequent investigations should contemplate evaluating the enduring ramifications and the most suitable dosage schedule of syringic acid in the context of priapism. Furthermore, this study did not have the capacity to demonstrate the effect of syringic acid on cavernosal tissue in healthy rats, a dimension that could have provided valuable comparative insights. Finally, the absence of immunohistochemical analyses in the pathological assessments of this study limited the researchers' ability to investigate specific cellular markers and molecular pathways. These limitations highlight areas for improvement and potential avenues for future research when studying the effects of syringic acid on cavernosal tissue.

## Conclusions

In this study, the administration of syringic acid was found to alleviate tissue damage in cavernosal tissue resulting from ischemia-reperfusion. It was observed that serum levels of antioxidants, including SOD and GSH-px, increased, accompanied by a decrease in MDA levels, a marker for lipid peroxidation. The proinflammatory cytokines TNF-alpha and IL 1-beta levels were also found to have increased significantly secondary to ischemia-reperfusion injury, but this increase was significantly decreased with syringic acid treatment. Furthermore, in rats subjected to syringic acid treatment, enhancements in pathological scores were evident, including vasocongestion, inflammation, desquamation, and edema in cavernosal tissue. According to the conclusions derived from this experimental study, the use of syringic acid appears to be a promising and viable alternative therapeutic option for alleviating the effects of ischemia-reperfusion injury following priapism interventions in individuals affected by ischemic priapism. To achieve a more thorough understanding of the molecular mechanisms that underlie the efficacy of syringic acid, it is crucial to conduct prospective, randomized, and controlled clinical studies in the future. Such studies will offer more robust evidence and lend further support to the findings obtained from our research.

## References

[1] Pryor J, Akkus E, Alter G (2004). Priapism. J Sex Med.

[2] Tay YK, Spernat D, Rzetelski-West K, Appu S, Love C (2012). Acute management of priapism in men. BJU Int.

[3] Pautler SE, Brock GB (2001). Priapism: prom Priapus to the present time. Urol Clin North Am.

[4] Evliyaoglu Y, Kayrin L, Kaya B (1997). Effect of allopurinol on lipid peroxidation induced in corporeal tissue by veno-occlusive priapism in a rat model. Br J Urol.

[5] Filho DW, Torres MA, Bordin AL, Crezcynski-Pasa TB, Boveris A (2004). Spermatic cord torsion, reactive oxygen and nitrogen species and ischemia-reperfusion injury. Mol Aspects Med.

[6] Huang YC, Harraz AM, Shindel AW, Lue TF (2009). Evaluation and management of priapism: 2009 update. Nat Rev Urol.

[7] Deng Y, Li RW, Yang YL, Weiss S, Smith PN (2022). Pharmacological prevention of renal ischemia-reperfusion injury in a rat model. ANZ J Surg.

[8] Sahin M, Baytaroglu C, Sevgili E (2022). Cardioprotective effect of cilostazol on ischemia-reperfusion injury model. Braz J Cardiovasc Surg.

[9] Rob MM, Hossen K, Iwasaki A, Suenaga K, Kato-Noguchi H (2020). Phytotoxic activity and identification of phytotoxic substances from Schumannianthus dichotomus. Plants (Basel).

[10] Chen J, Yang J, Ma L, Li J, Shahzad N, Kim CK (2020). Structure-antioxidant activity relationship of methoxy, phenolic hydroxyl, and carboxylic acid groups of phenolic acids. Sci Rep.

[11] Sanli O, Armagan A, Kandirali E (2004). TGF-beta1 neutralizing antibodies decrease the fibrotic effects of ischemic priapism. Int J Impot Res.

[12] Esterbauer H, Cheeseman KH (1990). Determination of aldehydic lipid peroxidation products: malonaldehyde and 4-hydroxynonenal. Methods Enzymol.

[13] Sun Y, Oberley LW, Li Y (1988). A simple method for clinical assay of superoxide dismutase. Clin Chem.

[14] Paglia DE, Valentine WN (1967). Studies on the quantitative and qualitative characterization of erythrocyte glutathione peroxidase. J Lab Clin Med.

[15] Levine RL, Garland D, Oliver CN (1990). Determination of carbonyl content in oxidatively modified proteins. Methods Enzymol.

[16] Erkanli Senturk G, Erkanli K, Aydin U, Yucel D, Isiksacan N, Ercan F, Arbak S (2013). The protective effect of oxytocin on ischemia/reperfusion injury in rat urinary bladder. Peptides.

[17] Capece M, Gillo A, Cocci A, Garaffa G, Timpano M, Falcone M (2017). Management of refractory ischemic priapism: current perspectives. Res Rep Urol.

[18] Reed-Maldonado AB, Kim JS, Lue TF (2017). Avoiding complications: surgery for ischemic priapism. Transl Androl Urol.

[19] El-Bahnasawy MS, Dawood A, Farouk A (2002). Low-flow priapism: risk factors for erectile dysfunction. BJU Int.

[20] Hudnall M, Reed-Maldonado AB, Lue TF (2017). Advances in the understanding of priapism. Transl Androl Urol.

[21] Martínez Portillo FJ, Jünemann KP (1999). New aspects in the treatment of priapism. Andrologia.

[22] Munarriz R, Park K, Huang YH, Saenz de Tejada I, Moreland RB, Goldstein I, Traish AM (2003). Reperfusion of ischemic corporal tissue: physiologic and biochemical changes in an animal model of ischemic priapism. Urology.

[23] Wu MY, Yiang GT, Liao WT (2018). Current mechanistic concepts in ischemia and reperfusion injury. Cell Physiol Biochem.

[24] Rashedinia M, Khoshnoud MJ, Fahlyan BK, Hashemi SS, Alimohammadi M, Sabahi Z (2021). Syringic acid: a potential natural compound for the management of renal oxidative stress and mitochondrial biogenesis in diabetic rats. Curr Drug Discov Technol.

[25] Abaza MS, Al-Attiyah R, Bhardwaj R, Abbadi G, Koyippally M, Afzal M (2013). Syringic acid from Tamarix aucheriana possesses antimitogenic and chemo-sensitizing activities in human colorectal cancer cells. Pharm Biol.

[26] Kolukcu E, Kilic S, Parlaktas BS, Erdemir F, Unsal V, Atılgan D, Uluocak N (2019). The effects of oxytocin on penile tissues in experimental priapism model in rats. Int Urol Nephrol.

[27] Uluocak N, Atılgan D, Erdemir F, Parlaktas BS, Yasar A, Erkorkmaz U, Akbas A (2010). An animal model of ischemic priapism and the effects of melatonin on antioxidant enzymes and oxidative injury parameters in rat penis. Int Urol Nephrol.

[28] Kölükçü E, Parlaktaş BS, Uluocak N, Deresoy FA, Katar M, Unsal V (2021). Dapsone can be a new treatment option for reducing the detrimental effect of priapism. J Health Sci Med.

[29] Karaguzel E, Bayraktar C, Kutlu O (2016). The possible protective effects of dipyridamole on ischemic reperfusion injury of priapism. Int Braz J Urol.

